# Sensitivity and specificity of the neutrophil-to-lymphocyte ratio for predicting mortality in patients with non-metastatic acral melanoma

**DOI:** 10.1016/j.jdin.2025.04.020

**Published:** 2025-09-16

**Authors:** Claudia Natali Cano Pallares, Carlos Daniel Sánchez Cárdenas, Zaira Estefani Ruelas Guzmán, Guevara Castillo, Enrique García Velázquez, Nancy Pulido Díaz

**Affiliations:** a Dermatology service, “La Raza” National Medical Center, P.° de las Jacarandas S/N, La Raza, Azcapotzalco, 02990, Mexico City, Mexico; b General Hospital of Cuautepec, Av. Emiliano Zapata #700 Colonia Cuautepec Barrio Alto, 07100, Gustavo A. Madero, Mexico City, Mexico

**Keywords:** melanoma, Mexico, mortality, neutrophil-lymphocyte ratio, sensitivity, specificity

*To the Editor:* Several studies have investigated the prognostic value of the neutrophil-to-lymphocyte ratio (NLR) in melanoma, but there is little information in the Mexican population, where acral melanoma is the most frequent.^1^, ^2^, ^3^, ^4^ A retrospective study of diagnostic tests was conducted in patients with nonmetastatic acral melanoma stages II and III from 2012 to 2022. Patients were divided into 2 groups: patients who died from nonmetastatic melanoma (30 patients) and patients who survived (60 patients). To calculate the NLR value, a blood sample taken on the first day of the histopathologic confirmation of malignant melanoma was considered and was obtained by dividing the total number of neutrophils by the total number of lymphocytes. Patients with systemic infections, autoimmune diseases, and other neoplasms were excluded. Survival time and clinical-histopathologic characteristics were identified in the records. The cut-off point of the NLR was determined with the receiver operating characteristic curve. The diagnostic capacity values were determined. Subsequently, the Cox regression analysis and survival curves were performed and included topography, Breslow thickness (mm), ulceration, number of mitoses per mm^2^, vascular and neural invasion, age (years), sex, and NLR value of >2. An NLR cut-off point of 2 (area under the curve value, 0.901; 95% CI, 0.828-0.973; *P* = .000) (Fig 1, *A*) was determined, with a sensitivity of 90.2%, specificity of 46.6%, positive predictive value of 46%, negative predictive value of 90%, positive likelihood ratio of 1.69 (95% CI, 1.3-2.2), and negative likelihood ratio of 0.21 (95% CI, 0.07-0.64). The significant risk factors for patient mortality included an NLR value > 2 (hazard ratio [HR], 4.13; 95% CI, 1.2-13.9; *P* = .022) and Breslow thickness (HR, 1.49; 95% CI, 1.2-1.7; *P* = .000), as indicated in Fig. 1, *B*, with a corresponding 5-year survival rate of 31%. Ding et al^1^ determined the prognostic value of NLR in patients with melanoma; a significant decrease in the total survival rate was observed in patients from Western countries with an HR value of 2.34 (95% CI, 1.59-3.44; *P* < .001), whereas Asian countries showed an HR value of 2.35 (95% CI, 1.59-3.49; *P* < .001). The authors concluded that an elevated NLR value is useful in predicting poor outcomes of total survival rate in patients with melanoma. The most used cut-off point of the NLR value was 5.^1^ Ertekin et al^3^ determined the prognostic value of the NLR and platelet-to-lymphocyte ratio (≥2.1; HR, 1.30; 95% CI, 1.06-1.60; *P* = .013 and ≥ 184; HR, 1.37; 95% CI, 1.06-1.76; *P* = .014, respectively) and concluded that both are independent predictors for the prognosis of melanoma. The NLR HR result of Ertekin et al^3^ was less than that of our study. Lino-Silva et al^5^ determined the overall survival rate of patients with nonmetastatic melanoma and its association with NLR. They determined an NLR value with a cut-off point of ≥2. The 5-year overall survival rate was lower in patients who had an NLR value of ≥2 (63% vs 53%).^5^ In our study, the cut-off point was lower, with high sensitivity for predicting mortality, unlike the study of Ding et al.^1^ In addition, we found 4 times the mortality probability. We found the same cut-off point as the Lino-Silva et al^5^ study, with a 5-year survival rate of 31%. In conclusion, NLR value is a favorable prognostic factor for mortality in patients with nonmetastatic acral melanoma in stages II and III. This tool is useful, cost effective, and easy to perform

**Fig 1:**
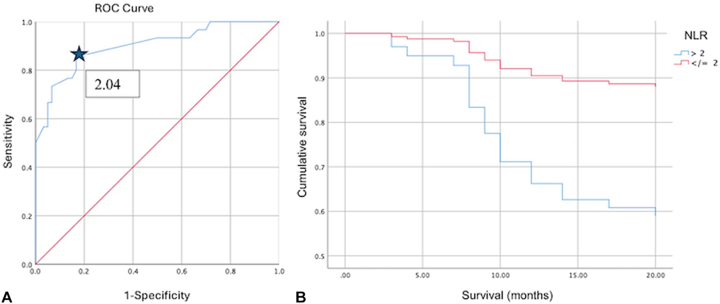
**A,** Area under the curve and cut-off point of the neutrophil-to-lymphocyte ratio of patients with nonmetastatic acral malignant melanoma stages II and III. **B,** Cox regression of patients with lymphocyte-to-neutrophil ratio with nonmetastatic acral malignant melanoma stages II and III.

## Conflicts of interest

None disclosed.
